# Estimating *Plasmodium falciparum* Transmission Rates in Low-Endemic Settings Using a Combination of Community Prevalence and Health Facility Data

**DOI:** 10.1371/journal.pone.0042861

**Published:** 2012-08-22

**Authors:** Joshua Yukich, Olivier Briët, Michael T. Bretscher, Adam Bennett, Seblewengel Lemma, Yemane Berhane, Thomas P. Eisele, Joseph Keating, Thomas Smith

**Affiliations:** 1 Department of Global Health Systems and Development, Tulane University School of Public Health and Tropical Medicine, New Orleans, Louisiana, United States of America; 2 Department of Public Health and Epidemiology, Swiss Tropical and Public Health Institute, Basel, Switzerland; 3 University of Basel, Basel, Switzerland; 4 London School of Hygiene and Tropical Medicine, London, United Kingdom; 5 Addis Continental Institute of Public Health, Addis Ababa, Ethiopia; London School of Hygiene and Tropical Medicine, United Kingdom

## Abstract

As some malaria control programs shift focus from disease control to transmission reduction, there is a need for transmission data to monitor progress. At lower levels of transmission, it becomes increasingly more difficult to measure precisely, for example through entomological studies. Many programs conduct regular cross sectional parasite prevalence surveys, and have access to malaria treatment data routinely collected by ministries of health, often in health management information systems. However, by themselves, these data are poor measures of transmission. In this paper, we propose an approach for combining annual parasite incidence and treatment data with cross-sectional parasite prevalence and treatment seeking survey data to estimate the incidence of new infections in the human population, also known as the force of infection. The approach is based on extension of a reversible catalytic model. The accuracy of the estimates from this model appears to be highly dependent on levels of detectability and treatment in the community, indicating the importance of information on private sector treatment seeking and access to effective and appropriate treatment.

## Introduction

Much of the malarious world has seen substantial scale up in intervention coverage in the last decade as a result of better funding for national malaria control programs [1]. Some programs are trying to reduce transmission with the long-term goal of interrupting it [2], but there is no generally applicable approach for measuring changes in transmission at low levels [3]. The entomological inoculation rate (EIR), estimated as a product of the sporozoite-positive host-seeking mosquitoes and the human-biting rate, is a definitive measure of transmission, but its measurement is technically challenging, labor intensive, provides only very imprecise estimates with low external validity, and is not feasible where EIR is low [4].

The incidence of new infections in the human population, also known as the force of infection (FOI), is a measure of transmission that is generally estimated without recourse to entomological measurements. The method of clearing parasites with an effective therapy and then following-up to observe when re-infection occurs is well established as an approach for measuring FOI in observational studies [5], [6] and intervention trials [7]–[9]. An alternative way of estimating FOI is by molecular typing of sequential samples of blood from exposed individuals [10]. Both of these methods require multiple field visits to a study cohort, and thus neither is cost-effective nor feasible as a routine monitoring approach. In low transmission settings, FOI can be estimated using serological approaches, making use of simple differential equation models to translate age-profiles of sero-positivity into a history of exposure [11]. These approaches are being used to compare transmission intensity across different settings [12], [13], but are of limited value in tracking rapid changes in transmission.

Longitudinal patterns of clinical incidence clearly provide most of the accessible information with which to track such changes, largely because of the relative abundance of annual parasite index (API) data through routine health information systems. In fact, in many parts of the world with low levels of transmission, especially outside Africa, the only malariometric data routinely collected are health facility-based clinical incidence (API) data. These data provide no information about sub-clinical infections, home treated infections, or infections among those not seeking treatment within the health system and can not be used directly to estimate transmission intensity.

In many settings, levels of malaria endemicity are assessed using parasite prevalence derived from population-based household surveys. In most infectious diseases, transmission can be measured by the incidence of infection, which can be estimated as prevalence divided by the average duration of infection. For malaria, however, the same prevalence value can result from a wide range of EIR values [3], depending on biting densities of mosquitoes, variations in how long infections persist, and the frequency of treatment within the community. Box S1 describes common metrics used to measure malaria transmission. Further, duration of malaria infections is hard to measure, and depends strongly on treatment rates. As transmission is reduced, the sample size needed to accurately estimate parasite prevalence (or changes in prevalence) using household surveys increases, making it a poor way to measure endemicity or transmission [14].

In this paper we propose an approach using a combination of API and prevalence survey data to estimate FOI. The approach is based on extending a reversible catalytic model [15] previously used for modeling the infection and recovery process for *Plasmodium falciparum* malaria [16], [17]. The extension allows for 1) the effect of treatment on duration of infection and 2) the incorporation of imperfect detection of infections in sampled individuals, or detectability [18]. The effect of treatment, through impact on the duration of infection, alters the relationship between incidence and prevalence. Detectability is most likely to be imperfect among individuals with asymptomatic infections [18], [19]. The extensions proposed here incorporate this bias in prevalence estimates, while remaining accessible and computable without the need for sophisticated software.

## Model

We use a reversible catalytic model of the relationship between prevalence and the force of infection, developed by Muench [15] and later adapted to malaria by Bekessy [16], which assumes that infections are distributed randomly over the population. The general form of the model is
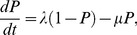
(1)where 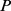 is (blood stage) parasite prevalence, 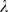 is FOI or incidence of new infections per person per time unit, and 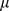 is the clearance rate of an infection per person per time unit (equal to the reciprocal of the average duration of an infection). Solving the above equation for 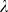 at equilibrium (where 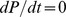) yields
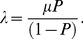
(2)This approach assumes that the equilibrium parasite prevalence in the population is a result of infection clearance and acquisition, and assumes prefect detectability of infection. Under the assumptions that having a pre-existing asymptomatic infection does not change the probability of acquiring a symptomatic infection, that only newly acquired infections can cause clinical cases, and that a proportion 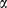 of these gets randomly, promptly, appropriately and effectively treated, without contributing to the parasite prevalence, the rate at which parasite negative individuals become positive is 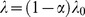, with 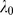 the FOI in the absence of treatment, corresponding to the rate at which infections are introduced. Data of 311 malaria therapy patients show that the first day of fever is 3 days (median) after the onset of detectable parasitaemia, with 98.7% less than 10 days, out of an average infection duration of about 200 days.

The average clearance rate 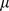 can be written 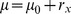, with 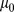 the natural clearance rate and 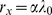 the (measurable) number of (new) infections receiving treatment per person per time unit, *i.e.* the treatment rate. Asymptomatic infections are thus naturally cleared with rate 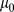, or when a new symptomatic super infection occurs which gets treated (clearing also the asymptomatic infection). This leads to:
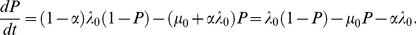
(3)Again assuming equilibrium, this leads to an estimate of the FOI of
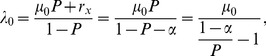
(4)with information on treatment included, and 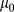 estimated from the duration of untreated infections.

When detectability 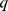
_,_ the probability of detecting an existing infection (in a cross-sectional survey), is incorporated into the model, an additional term 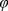
_,_ for the measured prevalence, is required. True prevalence, 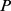
_,_ is related to measured prevalence, 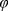
_,_ by the relationship 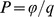.

With 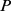 substituted, the model incorporating both treatment and detectability in the equilibrium state is
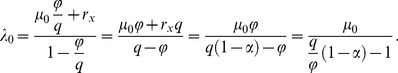
(5)This model considers all parasites in an infected host as equivalent, and all hosts as identical. Additionally, as this is an equilibrium model, it does not account for seasonality or trends, but it can be repeatedly estimated over time. Model parameters are summarized in Box S2.

**Figure 1:**
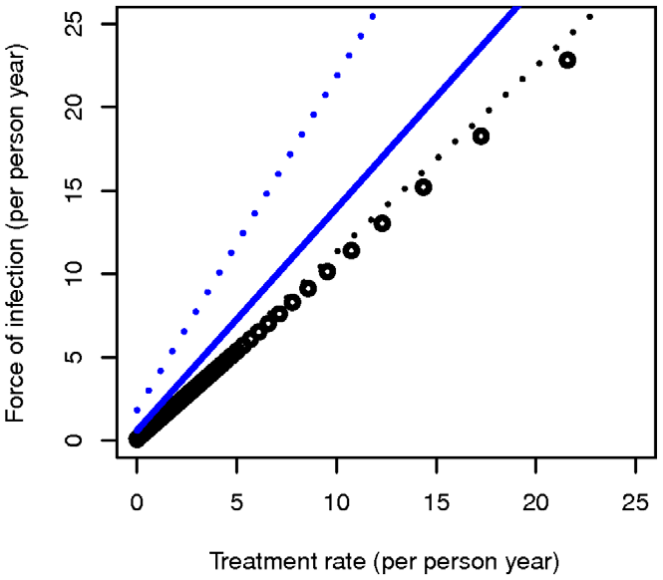
Force of infection against treatment rate. Force of infection (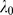: new infections per person year) against treatment rate (new infections treated per person year) at 5% measured prevalence (black lines) and 25% measured prevalence (blue lines), with perfect detectability (solid lines) and 50% detectability (dotted lines), with an average natural parasitaemia duration of 200 days.

In the limit when prevalence is zero (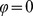
_)_ then the estimated force of infection is equal to the rate of treatment 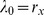. Figure 1 illustrates the relationship between FOI (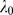) and treatment rate at 5% and 25% measured prevalence and an average natural parasitaemia duration of 200 days with perfect detectability and 50% detectability [18]. The difference in FOI at the two detectability levels is small at 5% measured prevalence, and larger at 25% measured prevalence. When detectability is at 50%, the FOI estimate is higher due to infections which are missed in cross sectional prevalence surveys. A higher treatment rate corresponds to higher incidence for the same observed prevalence, in-line with expectations.

**Figure 2:**
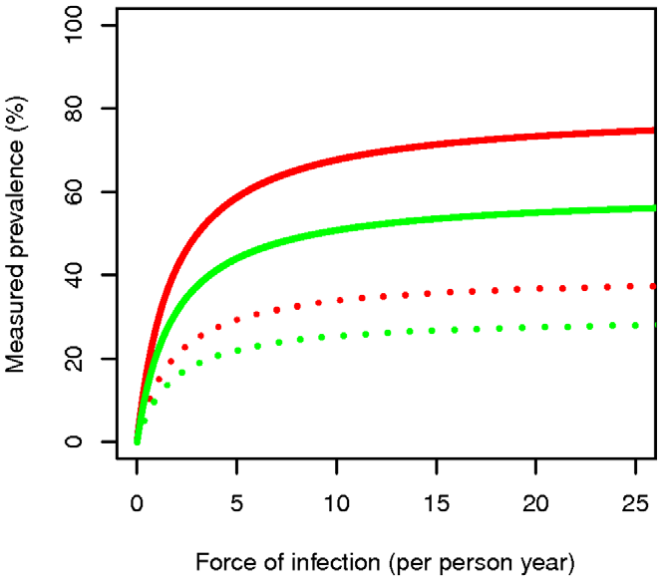
Measured prevalence against force of infection. Measured prevalence against force of infection (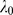: new infections prior to any treatment per person year) at 20% (red lines) and 40% (green lines) treatment rate of new infections; perfect detectability (solid lines) and 50% detectability (dotted lines), with an average natural parasitaemia duration of 200 days.

Figure 2 shows the relationship between the measured prevalence against FOI under two different assumptions about treatment; 20% or 40% of new infections receiving curative treatment. The curves have an asymptote at the measured prevalence 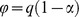
_._ For example, at 50% detectability of parasitaemia in cross sectional surveys and 20% of new infections treated, the model has an asymptote at a measured prevalence of 40%, while with 40% of new infections treated, measured prevalence is 30%. As expected, with 100% detectability, the observed prevalence values are exactly double that of the values at 50% detectability. Near the asymptote, the ability to estimate the FOI from the measured prevalence 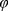 is limited.

**Figure 3:**
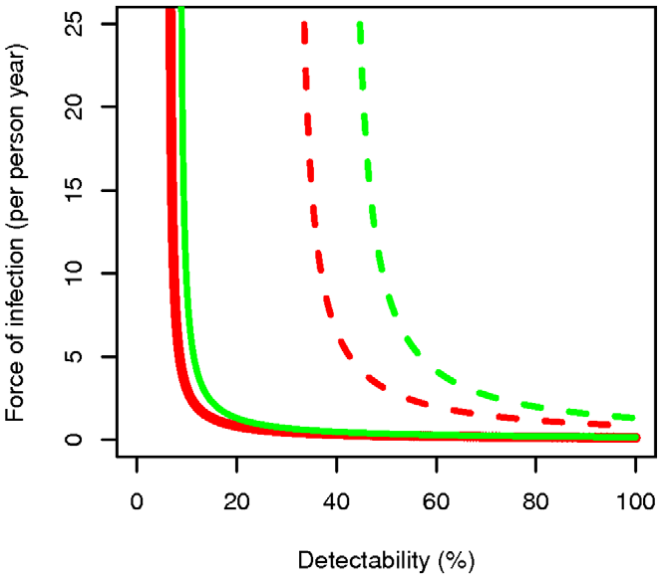
Force of infection against detectability of parasitaemia. Force of infection (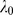: new infections prior to any treatment per person year) against the probability of detecting an existing parasitaemic infection (in a cross sectional survey) at 5% measured prevalence (solid lines) and 25% measured prevalence (dashed lines), with a treatment probability of new infections of 20% (red lines) and 40% (green lines), with an average natural parasitaemia duration of 200 days.

Figure 3 shows the relationship between the FOI (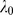) against the probability of detecting an existing parasitaemic infection (in a cross sectional survey) at two different measured prevalences; 5% or 25%. The curves have asymptotes at 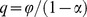 near to which the estimate of the FOI is very sensitive to the detectability.

## Model with Field Data

Data on treatment were derived from malaria epidemic detection surveillance sentinel sites in Ethiopia [20]–[22]. All sentinel sites have shown high (nearly perfect) testing rates among suspect cases and good adherence of providers to test results. Microscopy performance was variable across all sites, but in the two selected sites, concordance with expert readings was high (>90%). Over 1.5 years of observation at two sites, treatment rates were estimated based on API data from the sentinel health centers and treatment seeking behavior estimates from a malaria indicator survey [20]–[22] (adjustments were made to increase the number of treatments based on the fraction of patients who sought and received anti-malarials in the private sector *vs* those who sought care and received anti-malarials in the public sector). Estimates of parasite prevalence from the malaria atlas project [21] were used to estimate local parasite prevalence. Mean *P. falciparum* parasite rates children from age 2 to age 10 (*Pf*PR_2–10_) within a 10 km (2-cell) radius of the health center were used as an estimate of local malaria parasite prevalence. Tables 1 and 2 show the input values for each site.

**Table 1:** Monthly malaria treatments at Bulbulla and Asendabo health centers.

Year	Month	Bulbulla HC	Asendabo HC
2010	Apr	139	316
	May	297	288
	Jun	979	398
	Jul	331	222
	Aug	201	195
	Sep	95	166
	Oct	132	333
	Nov	89	135
	Dec	46	102
2011	Jan	35	57
	Feb	30	19
	Mar	27	11
	Apr	9	43
	May	28	78
	Jun	62	106
	Jul	94	261
	Aug	835	534
	Sep	243	293

**Table 2:** Other parameter values.

Model parameter	Input Value
Daily natural clearance rate	1/200
Detectability	50%
Observed prevalence in Bulbulla	7.2%
Proportion of antimalarial drugs obtained in private sector	65.7%
Catchment population Bulbulla	54,157
Observed prevalence Asendabo	10.4%
Catchment population Asendabo	48,111

**Figure 4:**
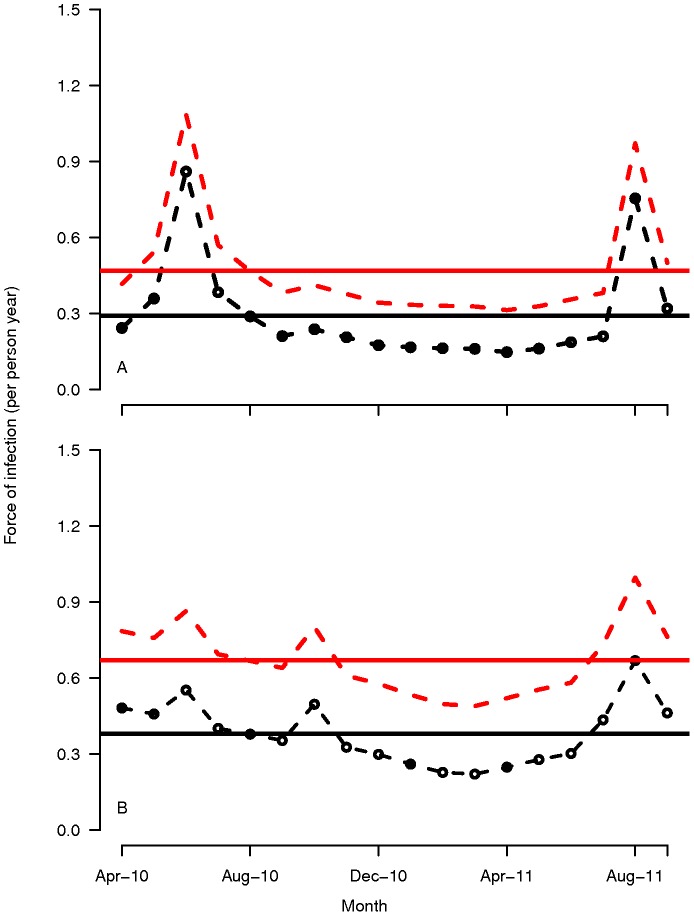
Monthly and average force of infection estimates for Bulbulla and Asendabo health center catchment areas in Ethiopia. Monthly (dashed lines) and annual average force of infection (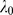: new infections prior to any treatment per person month) estimates assuming perfect detectability (black lines) or 50% detectability (red lines) in **A** Bulbulla and **B** Asendabo health center catchment areas.

Yearly equilibrium FOI by month and averages across the period for each Ethiopian sentinel site are shown in Figure 4.

This figure illustrates the sensitivity of measurement of FOI to treatment rates and model specification in relation to detectability, as the monthly estimates show that data from the dry period give low estimates of annual FOI with perfect detectability and FOI below 0.05 per person month assuming 50% detectability of infections. Spikes in FOI were seen during transmission periods at both sites, especially Bulbulla. Estimation of FOI with only data from high transmission periods would lead to erroneously high estimates of overall FOI for the entire period.

## Discussion

Force of infection for *P. falciparum* malaria can be estimated by combining API data and prevalence values from simple cross sectional surveys, with an extension of Muench's reversible catalytic model. This provides a straightforward low-cost measure of malaria transmission applicable in low transmission areas.

Catalytic models, which are much simpler than many other malaria transmission models, have long been used for estimating transmission rates from seroprevalence data [11], [23], [24]. They have also previously been used to estimate EIR and FOI from prevalence data obtained by microscopy [25]. However, these studies either ignored treatment or did not distinguish effects of treatment from those of natural infection clearance and imperfect detectability. Treatment seeking, especially when efficacious anti-malarials are available, dramatically affects any estimation of transmission rates from infection data because effective treatment substantially reduces the duration of infection [26], [27]. Increasingly, treatment is based on parasitological diagnosis using rapid diagnostic tests (RDTs) and the number of treatments in health facilities is being recorded in health management information systems (HMISs). The use of RDTs has increased the value of HMIS data, and in low transmission areas, API values derived from HMISs are often used as measures of malaria transmission.

However, HMISs remain an unreliable and incomplete source of treatment data [28] because they do not capture private sector or home treatment for malaria, and need to be augmented by population-based information on patients who seek treatment outside the formal public sector. If HMIS data are to be used to estimate transmission rates they also need to be augmented by population-based parasite prevalence data, which provide information on infections that have escaped treatment. Fortunately, recalls of treatment-seeking behavior and parasite prevalence data are both routinely collected in nationally representative malaria indicator surveys (MISs) repeated every two to three years in many endemic countries. The combination of MIS and HMIS data, using the algorithm described in this paper, thus provides a widely applicable means of estimating the FOI. One limitation of this approach is that data on treatment seeking derived from nationally representative surveys may fail to capture local variation. Furthermore, such data, which are usually available only semi-annually, will also fail to capture seasonal dynamics in treatment seeking. In many areas, systems for collection of HMIS data are weak and imprecise, and so would require strengthening before they can be considered a reliable source of treatment data.

This approach involves several approximations. It relies on equilibrium solutions, though in reality the system will generally not be at equilibrium. The simple model described in the main text also ignores the effects of super-infection, and does not explicitly incorporate multiplicities of infection greater than one. At high transmission levels, most hosts harbor many co-infecting clones, but allowing for super-infection leads to substantially more complicated models and requires an iterative approach for estimating the FOI from prevalence and API data (see File S1). Even these models with super-infection ignore further complications resulting from variations between hosts in levels of detectability, acquired immunity, levels of exposure and in access to treatment, all of which arise because of dependence between infections and which are therefore important at higher levels of transmission. At high incidences the same prevalence can thus result from a wide range of EIR values [29], depending on these sources of heterogeneity [30]. Where prevalence is above about 20%, a more detailed model capturing these effects would seem appropriate. Further work should include analyses of sensitivity to the assumptions about diagnostic performance, and more critical evaluation of the limits over which the approach is applicable.

At lower prevalence, allowing for super-infection makes little difference to the relationship of prevalence and FOI (see File S1 and Figures S1 and S2) and it appears as if it is reasonable to treat distinct infection events as independent and to account for treatment. However, the need to incorporate imperfect detection of parasites depending on the number of co-infections complicates the application of this model. Indeed, hosts with sub-patent infections are a greater proportion of the infected hosts at lower prevalence levels because a lower multiplicity of infection translates into a lower probability that there is at least one clone with a density above the detection level [18], [19].

Seasonality and age effects also need to be considered. Malaria transmission generally varies greatly by season, as illustrated by our Ethiopian example, so information collected at only one time of the year is likely to be unrepresentative, leading to biased estimates of FOI. Case-incidence is the main driver of seasonality in the estimates of FOI, so it is probably good enough to use annual averages for the prevalence and treatment-seeking behavior, but it is worrying that these data are usually collected only at one time-point which may be unrepresentative. Additionally, given the absence of diagnostics and widespread use of ineffective anti-malarials in much of the private sector, fluctuations in effective and appropriate treatment levels in the private sector do not necessarily track the patterns in the public sector [31]. Moreover, MISs mainly collect data from children less than five years of age, and both prevalence and treatment seeking behavior can vary by age and on small spatial scales. In our data example, we used data for two to ten year olds, a limitation of the approach, as compared to all ages prevalence data. All this adds to the case for MISs to be conducted in a rolling manner throughout the year [32], to be extended to include older people, and our model to be validated in a range of settings, including those where other estimates of FOI are available.

Despite all these reservations, estimating the FOI from a combination of case series and cross-sectional survey data remains a much more practicable means of tracking malaria transmission in programs than entomological assessment of the inoculation rate. The FOI and the EIR are closely related but not identical, because the FOI excludes bites by infectious mosquitoes that do not end up being infectious to humans [5], [33], [34], making FOI a more appropriate measure of transmission, while EIR is technically a measure of exposure. FOI is only indirectly related to burden of disease, which also depends on levels of clinical immunity, on promptness of treatment, and on the quality of care for severe disease. Measurement of disease burden is explored in a companion paper [35]. As malaria control programs, even in sub-Saharan Africa, increasingly re-orient themselves from reducing disease towards reducing transmission, there is a need for them to clearly distinguish between measures of disease burden and transmission, and estimating FOI may be the best avenue to accurately understand the level of transmission achieved. This requires comprehensive information on patient treatment rates from all care-providers as well as prevalence data, with allowance for imperfect detection of parasites.

## Conclusions

A reversible catalytic model that incorporates treatment and detectability can be used to estimate *P. falciparum* malaria FOI from HMIS and prevalence data from community surveys. The approach also requires an estimate of the proportion of effective treatments included in the HMIS out of all treatments and uses estimates from research studies of levels of detectability of parasites and of duration of untreated infections. This approach is much more straightforward than measuring the entomological inoculation rate. It is mainly applicable in low transmission settings, where there is a critical need for estimation of transmission rates when considering re-orientation of control programs towards elimination. Accurate results depend on availability of time series of numbers of treatments of confirmed cases from both private and public providers and an understanding of the levels of both patent and undetectable infections prevalent in an area.

## Supporting Information

Box S1 **Standard metrics of malaria transmission.** Adapted from Smith DL, Smith TA and Hay I; Chapter 7. Measuring Malaria for Elimination. in *A Prospectus for malaria elimination*. The Malaria Elimination Group: The Global Health Group UCSF Global Health Sciences (2009).
(DOCX)

Box S2 **Model Parameters and Inputs.**
(DOCX)

Figure S1 **Compartment models for effects of treatment on prevalence.** Model **A** corresponds to the model in the main text, which considers all parasites in an infected host as equivalent; Model **B** is an infinite server queuing model, where the effect of treatment is to remove only one infection at a time. The value of *n* is the number of concurrent co-infections (multiplicity of infection): only the first three infected categories are shown. Model **C** is a variant of the infinite server model, in which treatment removes all infections.
(TIF)

Figure S2 **Comparison of prevalence values predicted by different models.** The black dots each correspond to the means of 1000 simulations of the model illustrated in Figure **S1C**. The red lines to prevalence predicted by model **S1A**; the blue lines to that predicted by model **S1B**, and the black lines to the approximation in equation A11. In all cases a clearance rate of 0.005 per day was assumed.
(TIF)

File S1 **Models treating super-infections as independent of each other.**
(DOCX)
