# Supplementary material for: Estimating Plasmodium falciparum Transmission Rates in Low-Endemic Settings Using a Combination of Community Prevalence and Health Facility Data
Source: PLoS One. 2012 Aug 22;7(8):e42861. doi: 10.1371/journal.pone.0042861 (PMC3425560; doi:10.1371/journal.pone.0042861)
Supplement: Box S1 — Standard metrics of malaria transmission. Adapted from Smith DL, Smith TA and Hay I; Chapter 7. Measuring Malaria for Elimination. in A Prospectus for malaria elimination. The Malaria Elimination Group: The Global Health Group UCSF Global Health Sciences (2009). (DOCX) [file pone.0042861.s001.docx]

Parasite rate (**PR**) – The prevalence of non-infective asexual blood stage parasites. This varies with age and species of parasite.

Entomological inoculation rate (**EIR**) – The number of potentially infective mosquito bites experienced by one person during the course of one time period, typically one year. Usually estimated by counting the number of *Anopheles* *spp.* mosquito bites received per person during a fixed time interval (human biting rate) and measuring the proportion of *Anopheles* *spp.* mosquitoes caught with sporozoites present in their salivary glands, then taking the product of these numbers.

Force of infection (**FOI**) – The rate at which humans become infected by malaria. It can be estimated using sero-prevalence surveys or by artificially clearing a subset of humans of all parasites and measuring the time until reinfection.

Annual parasite index (**API**) – Defined as the total number of confirmed cases per year divided by the total population, usually expressed per thousand.

Vectorial capacity – The expected number of infectious bites which arise from all of the mosquitoes that bite an infected person on a single day.

Basic reproductive number (**R_0_**) – The number of infections in humans that would result from one introduced infected human, after one parasite generation, in an immune naïve population in the absence of control measures.

Reproductive number under control (**R_c_**) – The number of infected humans that would result from one introduced infected human, after one parasite generation, in the presence of control measures and immunity.
