# Supplementary material for: Estimating Plasmodium falciparum Transmission Rates in Low-Endemic Settings Using a Combination of Community Prevalence and Health Facility Data
Source: PLoS One. 2012 Aug 22;7(8):e42861. doi: 10.1371/journal.pone.0042861 (PMC3425560; doi:10.1371/journal.pone.0042861)
Supplement: Box S2 — Model Parameters and Inputs. (DOCX) [file pone.0042861.s002.docx]

**λ_0_** *– Force of infection* (See Box S1)

***P*** *– True prevalence – derived from cross sectional survey data (with perfect detectability assumed)*

***φ*** *– Observed prevalence – derived from cross sectional survey data (when imperfect detectability is assumed)*

***q*** *– Detectability – the probability that a true infection is identifiable by the employed diagnostic method in a cross sectional survey*

***r_x_*** *– Treatment rate – the numbers of appropriate, prompt and effective treatments delivered expressed per person and per unit time – can be derived from health facility data combined with cross sectional survey data*

***μ_0_*** *– Natural clearance rate – estimated based on malaria therapy data, assumed similar in all populations and age groups*

***API*** *– Annual parasite index* (See Box S1)

***Proportion of malaria drugs obtained in private sector*** *- used to adjust the treatment rate at formal health facilities to incorporate private sector treatment, obtained from cross sectional survey data.*
