# Supplementary figures and images for: Estimating Plasmodium falciparum Transmission Rates in Low-Endemic Settings Using a Combination of Community Prevalence and Health Facility Data
Source: PLoS One. 2012 Aug 22;7(8):e42861. doi: 10.1371/journal.pone.0042861 (PMC3425560; doi:10.1371/journal.pone.0042861)

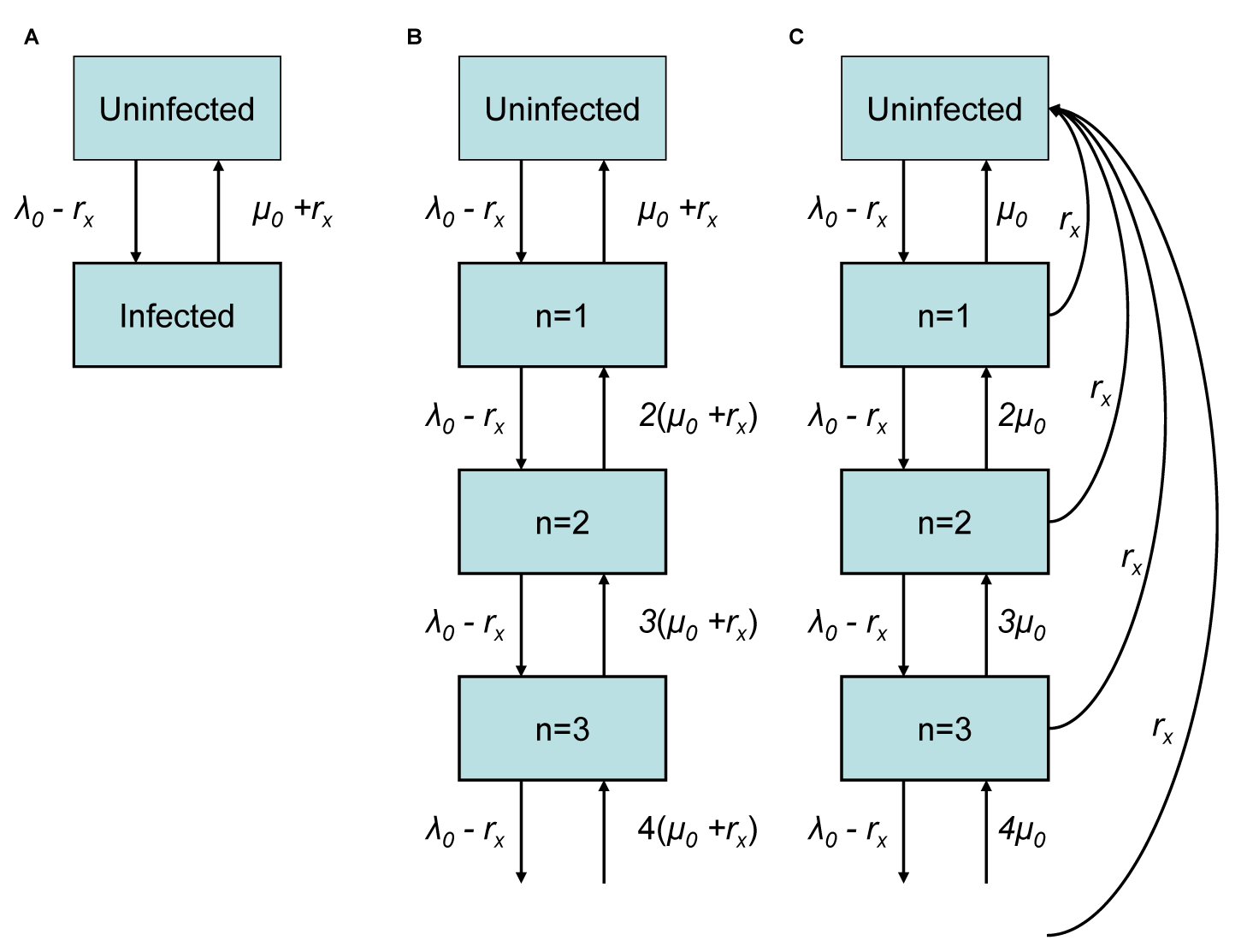

Supplement: Figure S1 — Compartment models for effects of treatment on prevalence. Model A corresponds to the model in the main text, which considers all parasites in an infected host as equivalent; Model B is an infinite server queuing model, where the effect of treatment is to remove only one infection at a time. The value of n is the number of concurrent co-infections (multiplicity of infection): only the first three infected categories are shown. Model C is a variant of the infinite server model, in which treatment removes all infections. (TIF) [file pone.0042861.s003.tif]

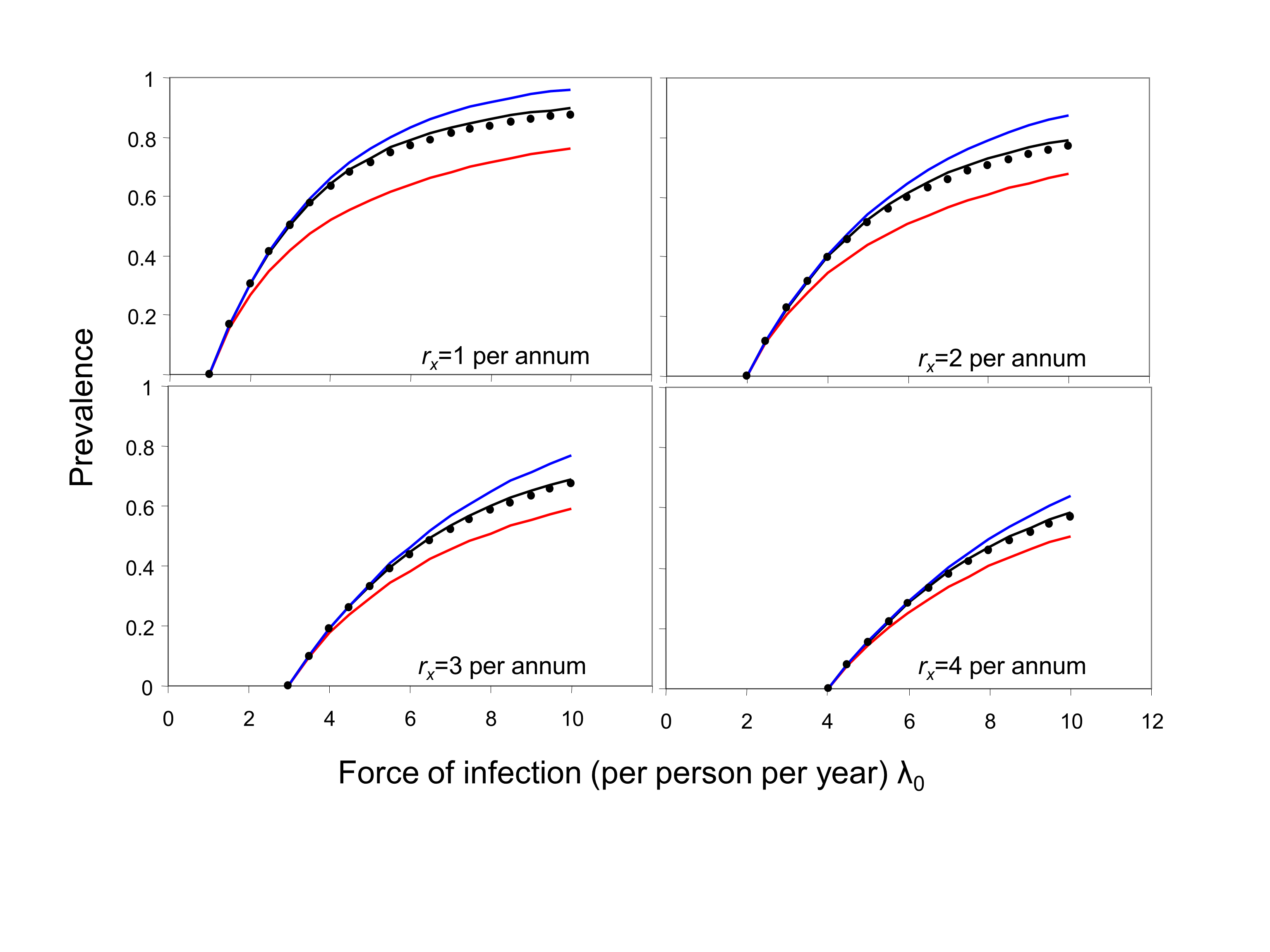

Supplement: Figure S2 — Comparison of prevalence values predicted by different models. The black dots each correspond to the means of 1000 simulations of the model illustrated in Figure S1C. The red lines to prevalence predicted by model S1A; the blue lines to that predicted by model S1B, and the black lines to the approximation in equation A11. In all cases a clearance rate of 0.005 per day was assumed. (TIF) [file pone.0042861.s004.tif]
