# Supplementary material for: Estimating Plasmodium falciparum Transmission Rates in Low-Endemic Settings Using a Combination of Community Prevalence and Health Facility Data
Source: PLoS One. 2012 Aug 22;7(8):e42861. doi: 10.1371/journal.pone.0042861 (PMC3425560; doi:10.1371/journal.pone.0042861)
Supplement: File S1 — Models treating super-infections as independent of each other. (DOCX) [file pone.0042861.s005.docx]

**Appendix A. Models treating super-infections as independent of each other**

The model described in the main text corresponds to the two-compartment model for the human population, shown in Figure S1A. This model considers all parasites in an infected host as equivalent, which we know not to be the case because of parasite genetic variation.

Treating super-infections as independent of each other, then, the infinite server queuing model in Figure S1B is correctly described by the ODEs:

$\frac{dp_{0}}{dt}=\left(\mu_{0}+r_{x}\right)p_{1}-\left(\lambda_{0}-r_{x}\right)p_{0}s$ and (A1)

$\frac{dp_{n}}{dt}=\left(\mu_{0}+r_{x}\right)\left(\left(n+1\right)p_{n+1}-np_{n}\right)+\left(\lambda_{0}-r_{x}\right)s\left(p_{n-1}-p_{n}\right)$, (A2), for $n>0$,

where $p_{n}$ is the proportion of the population with exactly $n$ (co-)infections and $s$ is the number of infections per infection event, which is assumed to be equal to 1.

The multiplicity of infection in individuals is described by the ordinary differential equation (ODE):

$\frac{dn}{dt}=\left(\lambda_{0}-r_{x}\right)s-\left(\mu_{0}+r_{x}\right)n=\left(1-\alpha\right)\lambda_{0}s-\left(\mu_{0}+\alpha\lambda_{0}\right)n$, (A3)

, and stable-state solutions:

$P=1-\exp\left(-\frac{\left(\lambda_{0}-r_{x}\right)}{\mu_{0}+r_{x}}\right)$, (A4)

$\lambda_{0}=r_{x}-\left(\mu_{0}+r_{x}\right)\ln\left(1-P\right)=-\frac{\mu_{0}\ln\left(1-P\right)}{1+\alpha\left(\ln\left(1-P\right)-1\right)}$, (A5)

This model can be described graphically as the compartment model shown in Figure S1B, in which clearance of each infection, either naturally or by treatment occurs independently. This does not allow for the fact that treatments in reality simultaneously clear all co-infections (Figure S1C), which leads to dependency between the treatments of different infections. The system described in Figure S1C is correctly described by the ODEs:

$\frac{dp_{0}}{dt}=p_{1}\mu_{0}+\left(1-p_{0}\right)r_{x}-\left(\lambda_{0}-r_{x}\right)p_{0}=p_{1}\mu_{0}-p_{0}\lambda_{0}+r_{x}=p_{1}\mu_{0}+\lambda_{0}\left(\alpha-p_{0}\right)$ and (A6)

$\frac{dp_{n}}{dt}=\left(n+1\right)p_{n+1}\mu_{0}-p_{n}r_{x}+\left(\lambda_{0}-r_{x}\right)p_{n-1}=\left(n+1\right)p_{n+1}\mu_{0}-\lambda_{0}\left(\left(1-\alpha\right)p_{n-1}-\alpha p_{n}\right)$, (A7), for $n>0$.

Unfortunately, this system of equations is not readily solved to give an expression for the equilibrium prevalence.

An approximate solution can be obtained by noting that from [1], the mean duration of a continuous period of infection (treating super-infections as independent of each other) in the model in Figure S1B, (in the absence of treatment, where models S1B and S1C are identical), at equilibrium is:

$T_{0}=\frac{\exp\left(\frac{\lambda_{0}}{\mu_{0}}\right)-1}{\lambda_{0}}$, (A8)

so the mean clearance rate of infection (the transition rate from the infected state with one or more infections to the uninfected state) is $1/T_{0}$. Similarly, the mean infection-free waiting time, once infections are cleared is $1/\lambda_{0}$.

In the presence of treatment, the average duration of a continuous period of infection if it is not treated is at equilibrium:

$T_{0}^{'}=\frac{\exp\left(\frac{\lambda}{\mu_{0}}\right)-1}{\lambda}$. (A9)

The transition rate from the infected to the uninfected state in the presence of treatment is therefore approximately (this relationship holds only approximately because the distribution of the duration of the infected state in this model is not exponential, and the clearance rate therefore varies with the age of the infection) $r_{x}+1/T_{0}^{'}$, and the mean duration of a continuous period of infection is therefore:

$T_{1}\approx\frac{1}{r_{x}+\frac{1}{T_{0}^{'}}}=\frac{1}{r_{x}+\frac{\lambda}{\exp\left(\frac{\lambda}{\mu_{0}}\right)-1}}$. (A10)

The assumption that new infections that are treated have zero duration, means that the mean infection-free waiting time between periods of infection is now $1/\lambda$. The expected prevalence allowing for treatment can be expressed as the expected proportion of time infected [1], and is thus approximately:

$P=\frac{T_{1}}{T_{1}+1/\lambda}\approx\frac{1}{1+\frac{1}{\exp\left(\frac{\lambda}{\mu_{0}}\right)-1}+\frac{r_{x}}{\lambda}}=\frac{1}{1+\frac{1}{\exp\left(\frac{\lambda_{0}-r_{x}}{\mu_{0}}\right)-1}+\frac{r_{x}}{\lambda_{0}-r_{x}}}=\frac{1}{1+\frac{1}{\exp\left(\frac{\lambda_{0}\left(1-\alpha\right)}{\mu_{0}}\right)-1}+\frac{\alpha}{1-\alpha}}$. (A11)

Unlike the solution for the model that considers all parasites in an infected host as equivalent, this cannot be rearranged to give an explicit formula for estimating $\lambda_{0}$ from the prevalence $P$ and treatment rate $r_{x}$. However, $\lambda_{0}$ can be obtained by solving numerically for given values of $P$, $r_{x}$ and $\mu_{0}$.

Figure S2 illustrates the comparison of prevalence values generated by the process in figure S1C, with those predicted by the different models including the approximation in equation (A11). At low levels of prevalence there is very little difference between the models. At higher levels of prevalence, the model that considers all parasites in an infected host as equivalent predicts the lowest prevalence, and the model that fails to allow for concurrent clearance on treatment, predicts the highest prevalence, while the approximation of equation (A11) gives very similar predictions of prevalence to those made by simulating the intended process.

The implications for the use of these models for estimating force of infection from observed treatment and prevalence data are that the model that considers all parasites in an infected host as equivalent would give force of infection estimates that are slightly biased upwards; the model of equations (A1)-(A5) would give estimates that are slightly biased downwards. The model of equation (A11) performs best, but the improvement over the other models is probably small. Further, equation (A11) has the significant disadvantage that it does not provide an explicit function for the force of infection, and so is more difficult to use in practice than the other algorithms.

**References**

1. Dietz K, Molineaux L, Thomas A (1974) A malaria model tested in the African savannah. Bull World Health Organ 50: 347-357.
